# Physiological and Molecular Responses to Variation of Light Intensity in Rubber Tree (*Hevea brasiliensis* Muell. Arg.)

**DOI:** 10.1371/journal.pone.0089514

**Published:** 2014-02-27

**Authors:** Li-feng Wang

**Affiliations:** Key Laboratory of Biology and Genetic Resources of Rubber Tree, Ministry of Agriculture, State Key Laboratory Incubation Base for Cultivation and Physiology of Tropical Crops, Rubber Research Institute, Chinese Academy of Tropical Agricultural Sciences, Danzhou, Hainan, China; US Naval Reseach Laboratory, United States of America

## Abstract

Light is one of most important factors to plants because it is necessary for photosynthesis. In this study, physiological and gene expression analyses under different light intensities were performed in the seedlings of rubber tree (*Hevea brasiliensis*) clone GT1. When light intensity increased from 20 to 1000 µmol m^−2^ s^−1^, there was no effect on the maximal quantum yield of photosystem II (PSII) photochemistry (Fv/Fm), indicating that high light intensity did not damage the structure and function of PSII reaction center. However, the effective photochemical quantum yield of PSII (Y(II)), photochemical quenching coefficient (qP), electron transfer rate (ETR), and coefficient of photochemical fluorescence quenching assuming interconnected PSII antennae (qL) were increased significantly as the light intensity increased, reached a maximum at 200 µmol m^−2^ s^−1^, but decreased from 400 µmol m^−2^ s^−1^. These results suggested that the PSII photochemistry showed an optimum performance at 200 µmol m^−2^ s^−1^ light intensity. The chlorophyll content was increased along with the increase of light intensity when it was no more than 400 µmol m^−2^ s^−1^. Since increasing light intensity caused significant increase in H_2_O_2_ content and decreases in the per unit activity of antioxidant enzymes SOD and POD, but the malondialdehyde (MDA) content was preserved at a low level even under high light intensity of 1000 µmol m^−2^ s^−1^, suggesting that high light irradiation did not induce membrane lipid peroxidation in rubber tree. Moreover, expressions of antioxidant-related genes were significantly up-regulated with the increase of light intensity. They reached the maximum expression at 400 µmol m^−2^ s^−1^, but decreased at 1000 µmol m^−2^ s^−1^. In conclusion, rubber tree could endure strong light irradiation via a specific mechanism. Adaptation to high light intensity is a complex process by regulating antioxidant enzymes activities, chloroplast formation, and related genes expressions in rubber tree.

## Introduction

Light is an important source of photosynthetic apparatus for photosynthesis and carbohydrate assimilation in plants, alga, etc. All photosynthetic organisms have to balance the efficient acquisition of light energy (light-harvesting) with protection from the adverse effects of light (photo-protection) [1]. In plants, chloroplasts capture light energy and causes accumulation of reactive oxygen species (ROS). In addition, ROS are also generated in other organisms accompanying biochemical reactions, mainly in mitochondria, peroxisomes or vacuoles. ROS include the superoxide anion (O_2_·^−^), hydrogen peroxide (H_2_O_2_), and the extremely short-lived hydroxyl radical (OH^·^). If not efficiently scavenged, these accumulated ROS will inevitably damage DNA, RNA, proteins, and lipid in organisms. To cope with elevated levels of ROS, aerobic cells have evolved a range of non-enzymatic and enzymatic antioxidant systems. For example, ascorbate (vitamin C)-glutathione cycle, superoxide dismutase (SOD), catalase (CAT), peroxidase (POD) enzyme system, tocopherol (vitamin E), and carotenoids (xanthophyll cycle pigments) are commonly known as antioxidants [2], [3]. Moreover, all photosynthetic organisms regulate the synthesis of their photosystem in response to changes of environment. Plants regulate both chloroplast and nuclear gene expression in response to photosynthesis mediated changes in cellular redox [4], [5]. Redox regulation of photosystem genes allows plants to ‘fine-tune’ synthesis of the photosystem in response to the light intensity.

Natural rubber is found in the latex of rubber tree (*Hevea brasiliensis* Muell. Arg.) and consists of high molecular weight cis-polyisoprene produced from the isoprenoid pathway [6]. Rubber tree, which originated in South America Amazon River Basin, is a typical tropical rainforest plant in favor of environment with high temperature and humidity [7]. However, rubber yield, biomass production, water use efficiency, and the associated physiological traits varied in different *Hevea* clones. Significant differences were observed in photosynthetic rate in different clones of rubber tree [8]. The relationship between photosynthetic capacity and development and growth of rubber tree was preliminary studied [9], [10]. These results showed great importance in rubber clone selection and breeding program for elite latex-timber clone. Recently, the development of molecular biology in rubber tree had revealed molecular mechanisms of natural rubber biosynthesis and brought new insights for regulation of natural rubber biosynthesis through different signal pathway. For instance, *HbCOA*, a long-chain-fatty-acyl-CoA reductase gene (EC 1.2.1.50), was found to play an important role in natural rubber biosynthesis [11]. *HbAPX*, an ascorbate peroxidases gene, was involved in antioxidant system [12]. *HbATP*, encoding the β-subunit of mitochondrial ATP synthase *in Hevea brasiliensis* was cloned [13]. *HbCuZnSOD* and *HbMnSOD* encoded antioxidant superoxide dismutase enzymes, and their roles in drought response were confirmed in rubber tree [14]. However, until this day, none of study was reported to reveal the relationship between the expression of these ROS-related genes and photosynthetic rate in rubber tree under different light intensities.

The purpose of this study is to delineate the effects of different light intensities on photosynthesis, assess the ROS contents, antioxidant enzymes activities and representative genes expressions in rubber tree. Our data indicated that increase in light intensity induced productions of ROS and expressions of ROS-related genes, which were highly related to the unique mechanism in response to high light irradiation in rubber tree.

## Results

### The effect of light intensity on photosystem II (PSII) photochemistry

**Figure 1:**
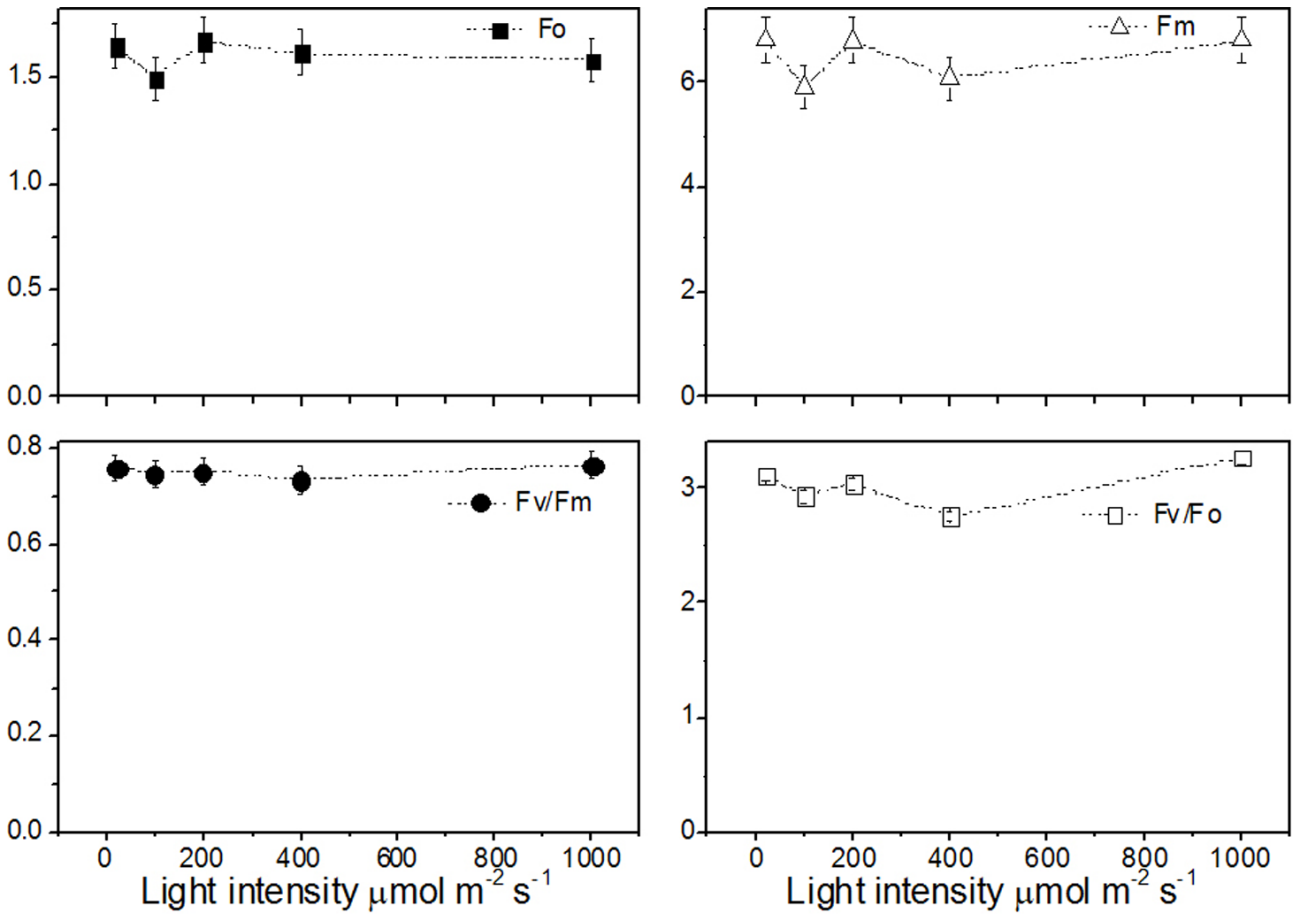
Effect of light intensity on the minimum fluorescence yield (Fo), the maximum PSII fluorescence yield (Fm), the maximum quantum yield of PSII photochemistry (Fv/Fm), and potential PSII activity (Fv/Fo) in seedlings leaves of rubber tree clone GT1. Values represent the mean ±SD of 6 replicate samples tested in replicate.

The changes in PSII photochemistry, utilization and dissipation of excess excitation energy in rubber tree clone GT1 leaves under different light intensities were systematically investigated. Figure 1 showed the changes in the minimum fluorescence yield (Fo), the maximum fluorescence yield (Fm), the maximum quantum yield of PSII photochemistry (Fv/Fm), and potential PSII activity (Fv/Fo) under different light intensities, respectively. The change in Fv/Fm were examined to determine the effect of different light intensities on the potential efficiency of PSII photochemistry. As light intensity increased, the Fv/Fm was fluctuated from 0.7–0.8 but showed no significant change under different light intensities. Similar result was found in Fv/Fo, and a slight variation was observed under different light intensities. Both Fo and Fm were decreased at 100 µmol m^−2^ s^−1^, but increased at 200 µmol m^−2^ s^−1^. Still, there was no significant difference present in the two values under 20 and 1000 µmol m^−2^ s^−1^. The Fv/Fm has been widely used as an indicator of photoinhibition [15]. These results suggested that no photoinhibition was occurred even at high light intensity of 1000 µmol m^−2^ s^−1^ in rubber tree seedlings.

**Figure 2:**
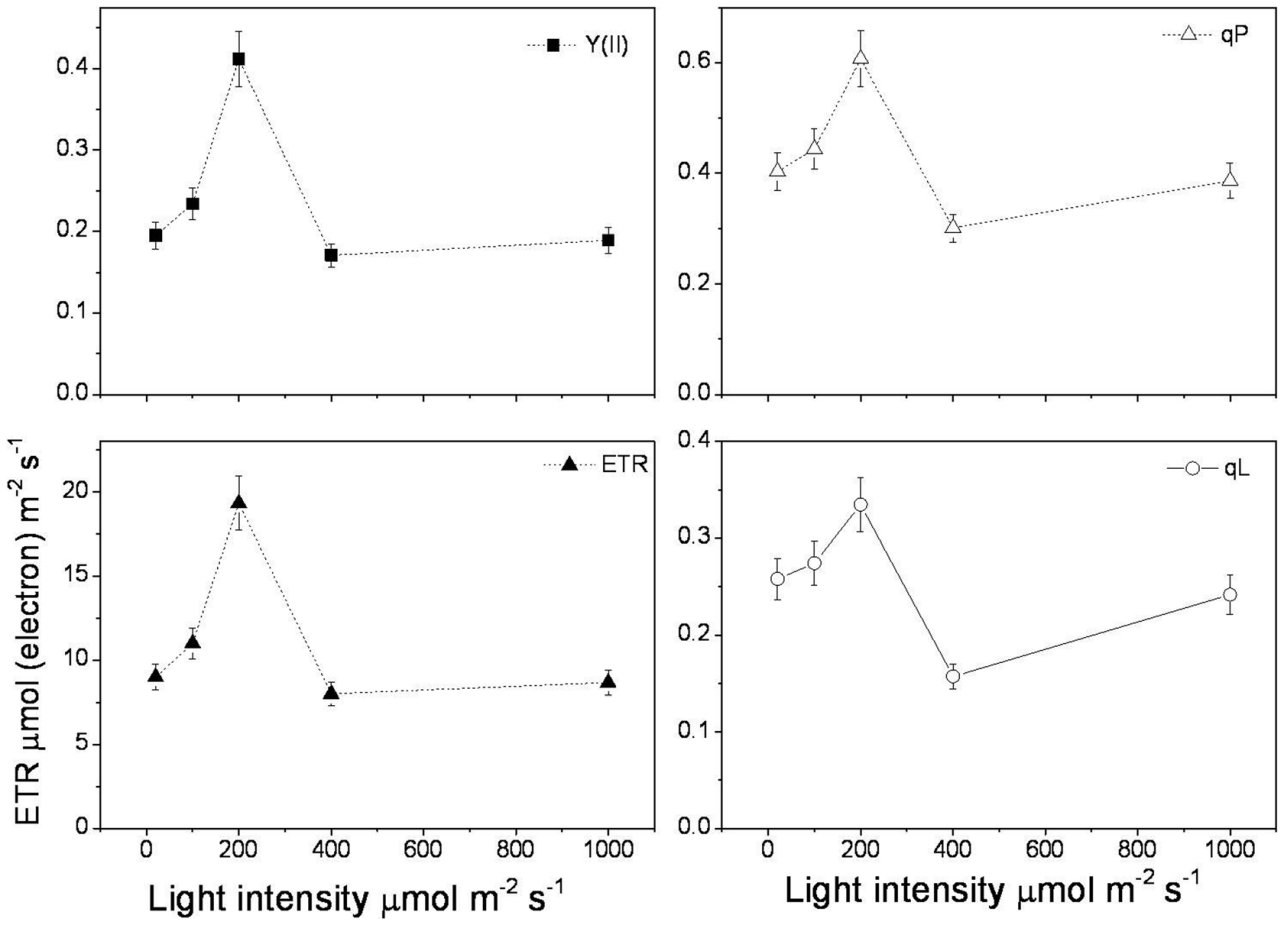
Effective photochemical quantum yield of PSII (Y(II)), photochemical quenching coefficient (qP), electron transport rate (ETR), and coefficient of photochemical fluorescence quenching assuming that all reaction centers share a common light-harvesting antenna (qL) measured under different light intensities in seedlings leaves of rubber tree clone GT1. Values represent the mean ±SD of 6 replicate samples tested in replicate.

**Figure 3:**
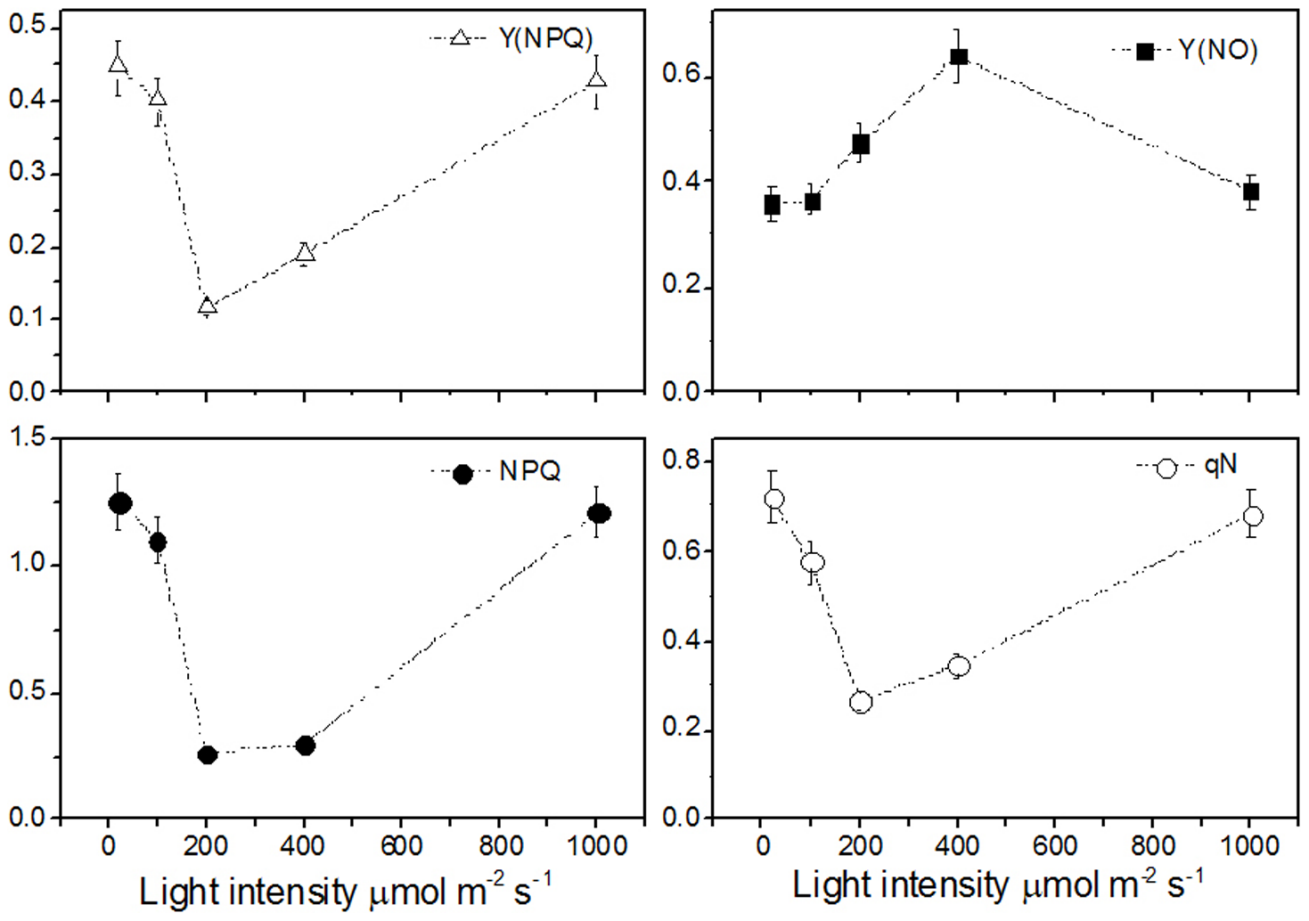
Effect of light intensity on quantum yield of non-photochemical energy conversion in PSII due to down-regulation of the light-harvesting function, Y(NPQ); quantum yield of non-photochemical energy conversion in PSII other than that caused by down-regulation of the light-harvesting function, Y(NO);and non-photochemical quenching coefficient, NPQ and qN; in seedlings leaves of rubber tree clone GT1. Values represent the mean ±SD of 6 replicate samples tested in replicate.

The effective photochemical quantum yield of PSII (Y(II)), photochemical quenching coefficient (qP), electron transfer rate (ETR), and coefficient of photochemical fluorescence quenching assuming interconnected PSII antennae (qL) were increased significantly as the light intensity increased, reached a maximum at 200 µmol m^−2^ s^−1^, but decreased from 400 µmol m^−2^ s^−1^ and held at a low level until 1000 µmol m^−2^ s^−1^ (Figure 2). These results suggested that the PSII photochemistry showed an optimum performance at 200 µmol m^−2^ s^−1^ light intensity. In order to identify the dissipation of excite energy, we measured the nonphotochemical quenching coefficient (NPQ), quantum yield of light-induced (ΔpH and zeaxanthin-dependent) non-photochemical fluorescence quenching (Y(NPQ)), and non-photochemical quenching of chlorophyll fluorescence (qN). We found that as the light intensity increase, Y(NPQ), NPQ, and qN were decreased sharply to reach a minimum at 200 µmol m^−2^ s^−1^, but increased from 400 to 1000 µmol m^−2^ s^−1^ (Figure 3). However, quantum yield of non-light induced non-photochemical fluorescence quenching (Y(NO)) showed a reverse pattern. It was markedly increased as the light intensity increased, reached a maximum at 400 µmol m^−2^ s^−1^, but decreased at 1000 µmol m^−2^ s^−1^ (Figure 3). The high level of Y(NO) at 400 µmol m^−2^ s^−1^ suggested photochemical energy transfer and heat could not quenching light energy absorbed by the seedlings leaves of rubber tree. The light harvest and transfer were broken in the donor side of PSII. Less light was transferred to the accept side for Q_A_ oxidation and following CO_2_ assimilation. When light intensity increased, the exciting light energy couldn't transfer to the PSII reaction center, but quenched at donor side of PSII, which resulted in the decreases of qP, qN, NPQ, and ETR at 400 µmol m^−2^ s^−1^ light intensity.

### The effect of light intensity on chlorophyll and β-carotene contents

**Figure 4:**
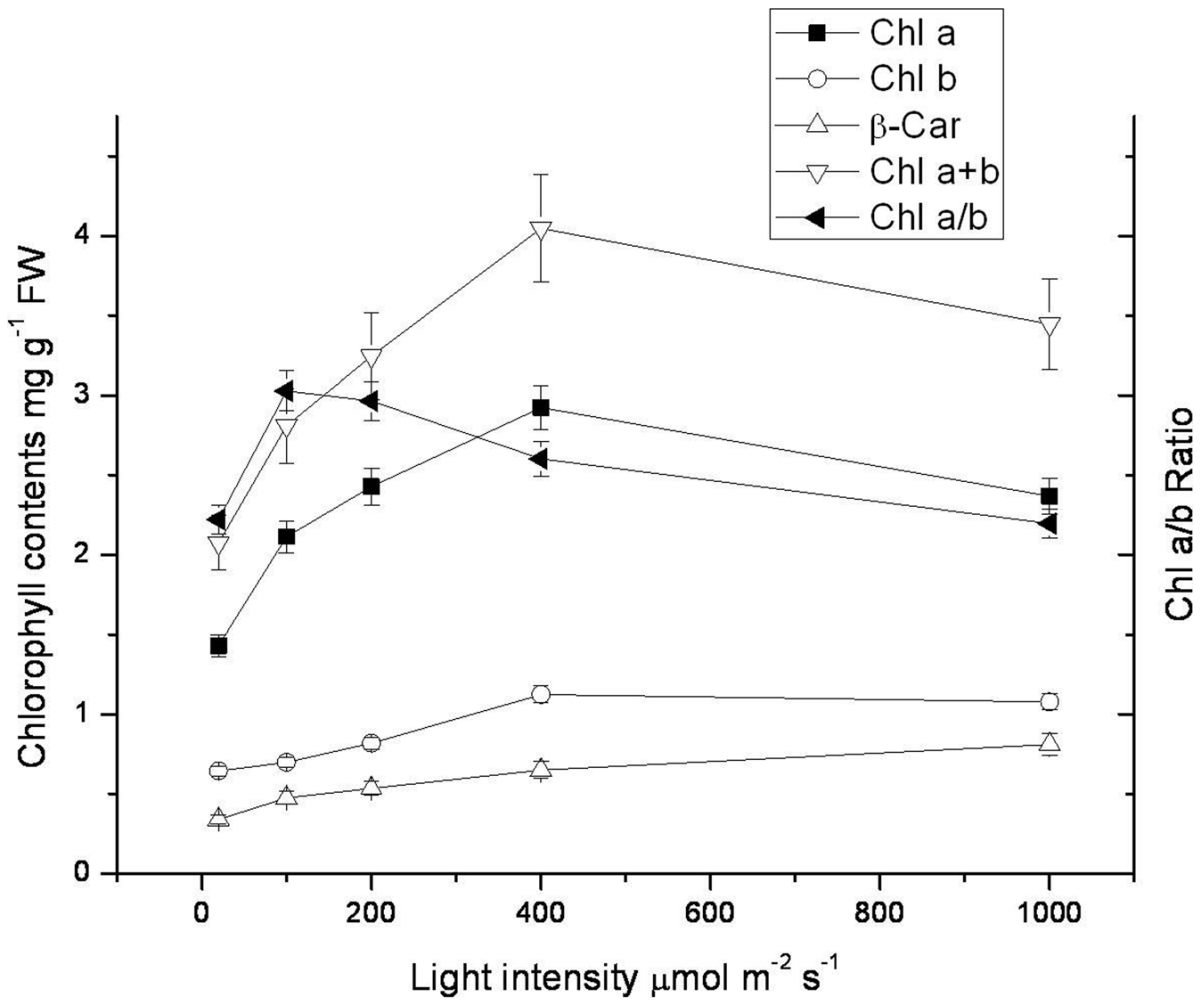
Effect of light intensity on chlorophyll contents in the seedlings leaves of rubber tree clone GT1. Values represent the mean ±SD of 6 replicate samples tested in replicate.

Chloroplast development needs formation of chlorophyll (Chl) and β-carotene. In order to find out whether exciting energy would be used as a light signal to regulating the chlorophyll and β-carotene synthesis, we measured the pigment composition under different light intensities. As showed in Figure 4, light significantly induced the total chlorophyll content (Chl a+b), and it reached the highest level at 400 µmol m^−2^ s^−1^. However, when light intensity increased to 1000 µmol m^−2^ s^−1^, the total chlorophyll content decreased slightly. Moreover, the increase rate of Chl a content was lower than that of Chl b, which resulted in the decrease of Chl a/b ratio from 100 to 1000 µmol m^−2^ s^−1^. These indicated rubber tree leaves in the dark green stage could endure strong light irradiation. A steadily increase was observed in the β-carotene content as the light intensity increase. These results showed different responses of chlorophyll and β-carotene to strong light irradiation. The β-carotene (xanthophyll cycle pigments) content was increased to scavenge the accumulated ROS and quenching light under high light intensity. The formation of chlorophyll was used to develop new chloroplasts. Since more light were absorbed by light harvesting chlorophyll protein complex, the formation rate of Chl b was higher than that of Chl a.

### Changes of physiological indices in response to alteration in light intensity

**Figure 5:**
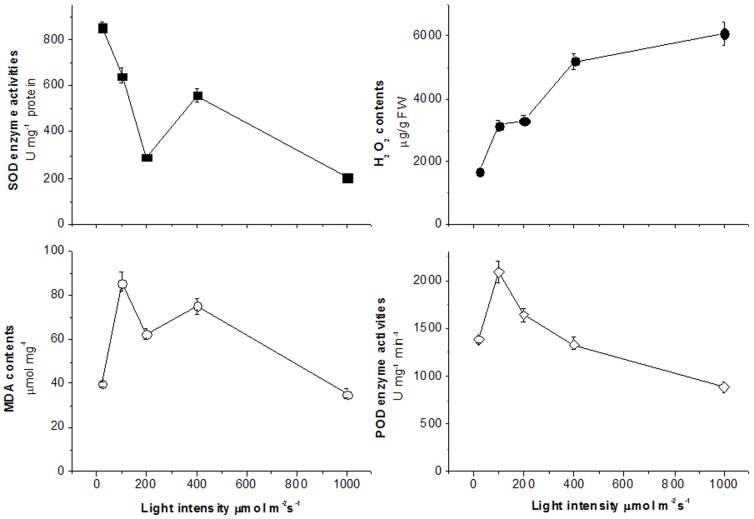
Effect of light intensity on MDA and H_2_O_2_ contents, and antioxidant enzymes activities in seedlings leaves of rubber tree clone GT1. Values represent the mean ±SD of 6 replicate samples tested in replicate.

Changes of physiological indices including the ROS and malondialdehyde (MDA) contents, and antioxidant enzymes activities were systematically analyzed under different light intensities. As showed in Figure 5, light directly induced oxidation reactions and increased the H_2_O_2_ content. Usually, the oxidative burst causes damage on lipid membranes. MDA is one of the most frequently used indicators of lipid peroxidation. Under stress conditions, the accumulation of MDA usually leads to the damage of cell membrane in plant and animal. As the light intensity increased, the MDA content underwent a complicated variation. It was increased at 100 µmol m^−2^ s^−1^, but decreased at 200 µmol m^−2^ s^−1^, then increased slightly at 400 µmol m^−2^ s^−1^, followed by a sharp decrease at the high light intensity of 1000 µmol m^−2^ s^−1^. Along with the increase of light intensity, the per unit activity of SOD enzyme was decreased dramatically. Meanwhile, the per unit activity of POD enzyme was increased at 100 µmol m^−2^ s^−1^, but decreased sharply from 200 µmol m^−2^ s^−1^. Since low light intensity was not strong enough to induce the formations of chlorophyll and antioxidant enzymes in rubber tree, MDA content was increased at 20 to 100 µmol m^−2^ s^−1^. These results indicated that high light intensity increased ROS contents, more antioxidant enzymes were formed and the amounts of enzymes were increased at the meantime, resulting in per unit activity of enzymes was decreased. So it did not induce membrane lipid peroxidation under high light intensity in rubber tree.

### Gene expression analysis under different light intensities

**Figure 6:**
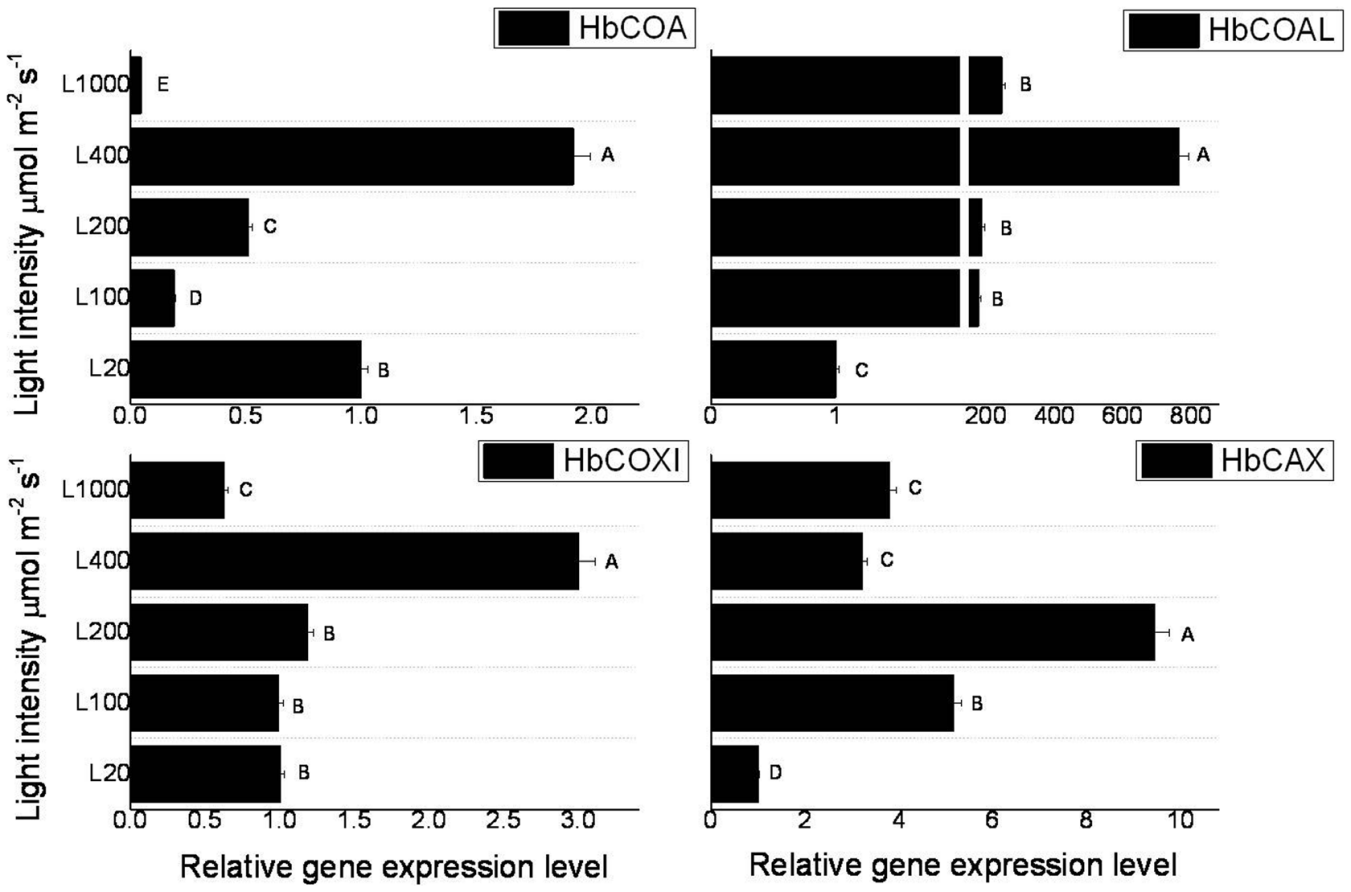
Effect of light intensity on the expressions of *HbCOA*, *HbCOAL*, *HbCOXI*, and *HbCAX* in seedlings leaves of rubber tree clone GT1. Values represent the mean ±SD of two biological replicates tested in triplicate. Bars with different letters show significant differences at the *P*<0.05 level.

**Figure 7:**
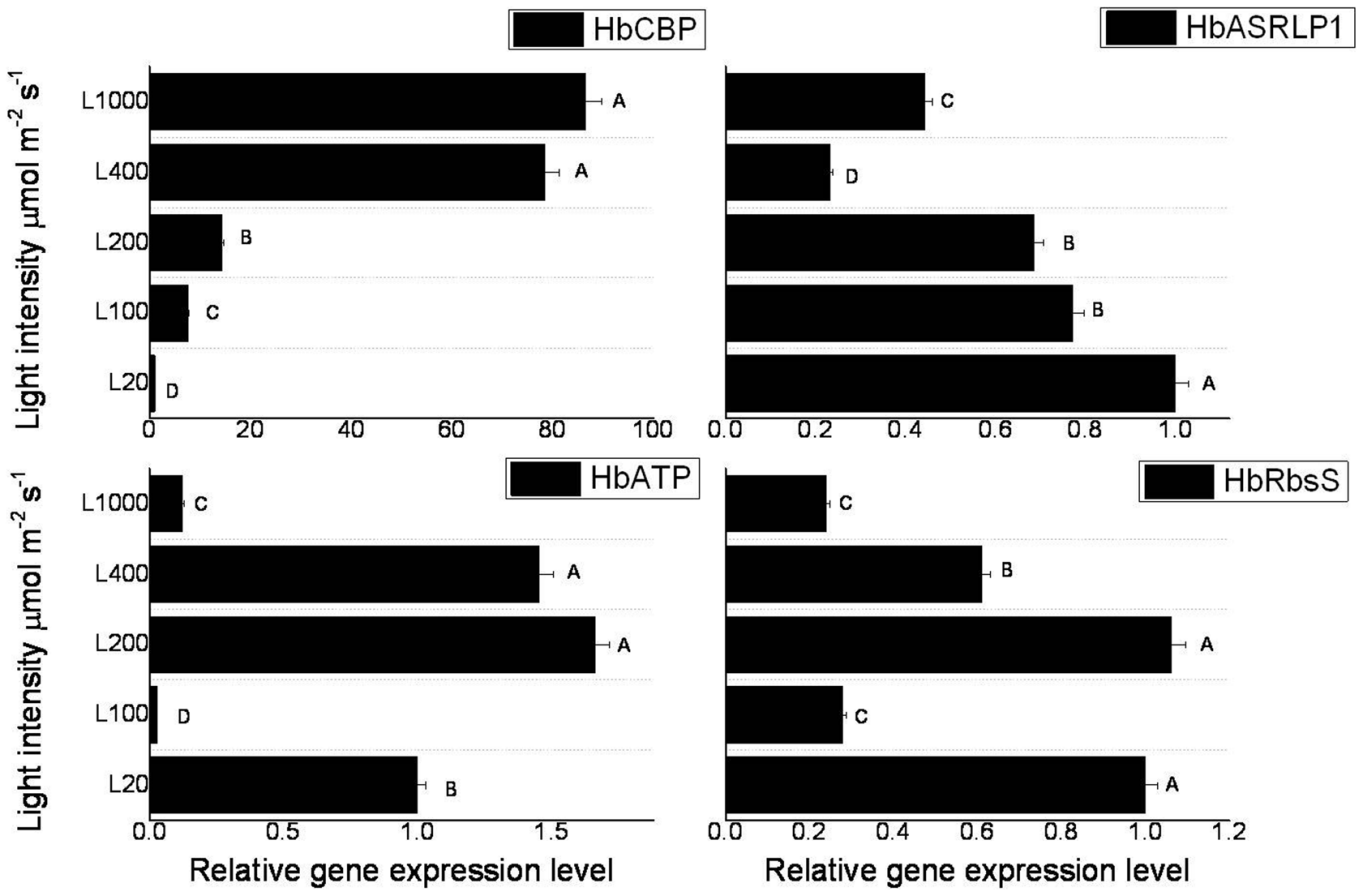
Effect of light intensity on the expressions of *HbCBP*, *HbASRLP1*, *HbATP*, and *HbRbsS* in seedlings leaves of rubber tree clone GT1. Values represent the mean ±SD of two biological replicates tested in triplicate. Bars with different letters show significant differences at the *P*<0.05 level.

Since light intensity affected photosynthesis and activities of antioxidant enzymes in rubber tree seedlings, the expression patterns of 12 genes located in mitochondria and chloroplasts were investigated comprehensively by real-time PCR. As showed in Figure 6, light intensity significantly induced the expressions of *HbCOA*, *HbCOAL* and *HbCOXI* with the same pattern. Their expressions reached the highest level at 400 µmol m^−2^ s^−1^, and increased 1.92-, 765-, and 3-fold comparing their expressions at 20 µmol m^−2^ s^−1^, respectively. For the three genes, this increase was followed by a strong decrease at 1000 µmol m^−2^ s^−1^. Transcript level of *HbCAX* was significantly up-regulated by the increase of light intensity, and reached the maximum level at 200 µmol m^−2^ s^−1^ (9.44-fold over the value at 20 µmol m^−2^ s^−1^) (Figure 6). With the increase of light intensity, the expression of *HbCBP* was continuously increased until 1000 µmol m^−2^ s^−1^ (Figure 7). However, *HbASRLP1* was continuously decreased (Figure 7). The *HbATP* transcript was firstly decreased at 100 µmol m^−2^ s^−1^, but increased slightly at 200 and 400 µmol m^−2^ s^−1^, and followed by a decrease at 1000 µmol m^−2^ s^−1^ (Figure 7). For the *HbRbsS*, encoding an enzyme involved in carbon fixation, its expression was firstly decreased at 100 µmol m^−2^ s^−1^, then increased to the normal level at 200 µmol m^−2^ s^−1^, and decreased from 400 to 1000 µmol m^−2^ s^−1^ (Figure 7). These suggested that high light intensity have diverse effects on the expressions of the mitochondria and chloroplasts genes. Mitochondria-related genes, i.e. at low light intensity, the transcripts of *HbCOXI* and *HbCAX* were increased as the light intensity increases, however, this trend was reversed beyond a certain level of light intensity. Most of these genes expressions were inhibited at high light intensity with 1000 µmol m^−2^ s^−1^, indicated that the mitochondria and chloroplasts, even rubber biosynthesis system would be inhibited by high light intensities.

**Figure 8:**
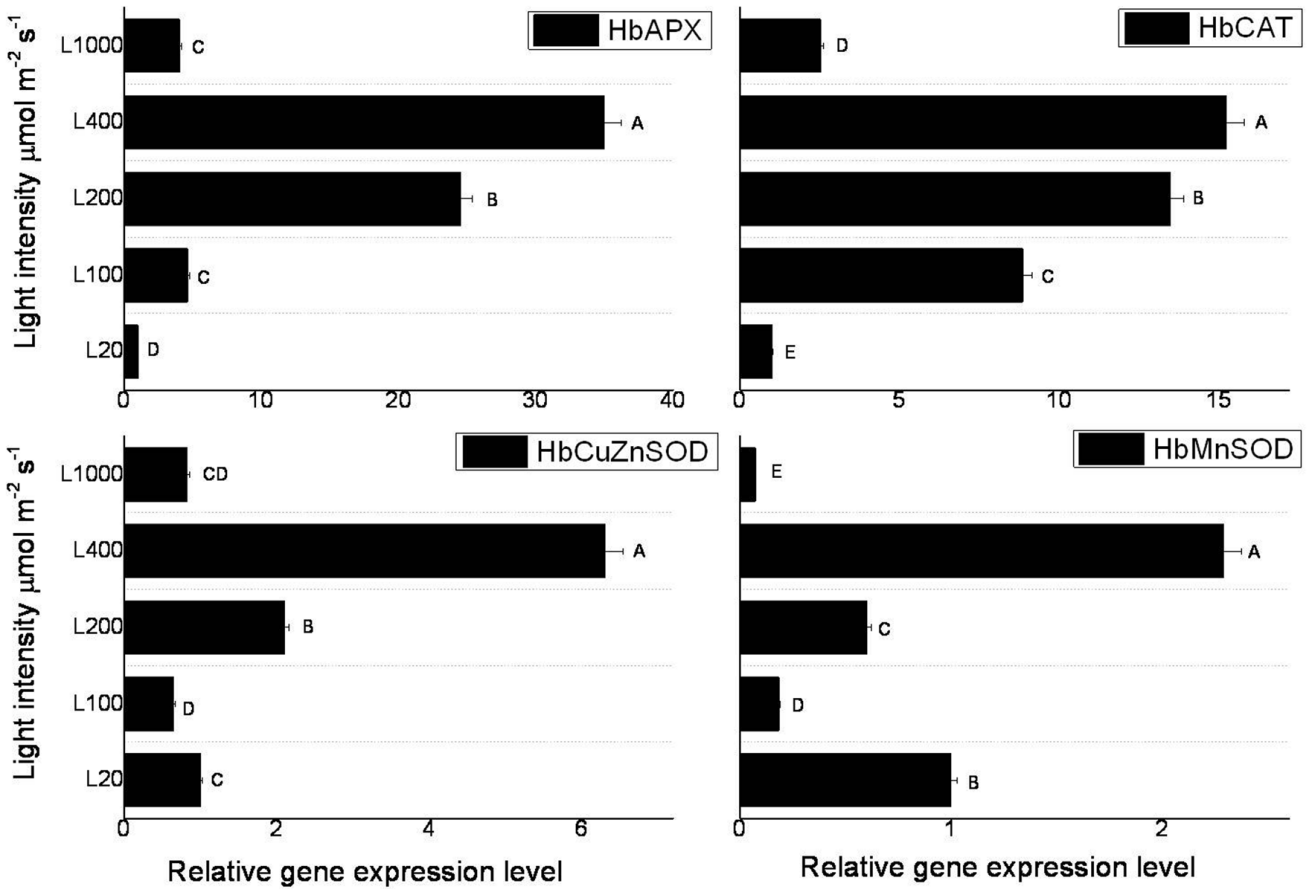
Effect of light intensity on the expressions of antioxidant-related genes in seedlings leaves of rubber tree clone GT1. Values represent the mean ±SD of two biological replicates tested in triplicate. Bars with different letters show significant differences at the *P*<0.05 level.

Light intensity significantly induced the expressions of antioxidant enzyme genes, i.e. *HbAPX*, *HbCAT*, *HbCuZnSOD*, and *HbMnSOD*. They reached a maximum at 400 µmol m^−2^ s^−1^, and increased 34.9-, 15.2-, 6.31-, and 2.29-fold over that at 20 µmol m^−2^ s^−1^, respectively. All of the four genes expressions were decreased at the light intensity of 1000 µmol m^−2^ s^−1^ (Figure 8). These suggested that the antioxidant enzymes took part in the response to the changes of light intensity. Under high light intensity, antioxidant enzymes genes expressions were up-regulated, and more antioxidant enzymes were formed to cope with the decrease of per unit activity of enzymes and increase of ROS.

## Discussion

Light had double effects on the growth and development of plants. On the one hand, a broad spectrum of light, ranging from UV-B to far-red light, affects every aspect of plant development, beginning with seed germination, vegetative growth, and reproductive development [16]. On the other hand, when plants are exposed to light intensities in excess of those that can be utilized in photosynthetic electron transport, nonphotochemical dissipation of excitation energy is induced as a mechanism for photoprotection of PSII [17]. The leaf development in rubber tree was usually classified in 3 stages, namely copper-brown, apple green, and the mature stage of dark green. During the rubber tree leaf ontogeny, the chlorophyll contents increase from less than 1.0 mg g^−1^ FW in copper-brown leaf to nearly 4 mg g^−1^ FW in dark green leaf, while Chl a/b ratio increase from 1.5 to 2.5 [18]. Light intensity induced the contents of chlorophyll as well as β-carotene as showed in this study. Since most Chl a is located in PSII reaction center, whilst Chl b is located in light harvesting chlorophyll-protein complex. The decrease of Chl a/b ratio suggested the balance between PSII reaction center and light harvesting chlorophyll-protein complex (Figure 4). The increase rate of Chl b was faster than that of Chl a, indicating that more light harvesting chlorophyll-protein complex were constructed to absorb more light. These were also found in the leaf development stages of rubber tree clone PB235 and RRIM600 [18].

The excess light energy usually quenched by photochemical reaction, nonphotochemical and thermal dissipation [19]. These induce complicit reaction in chloroplast, such as zeaxanthin formation [3], [20], reactive oxygen species signal pathway [21], and chloroplast acclimation [22]. In this study, besides the observation of the formation of chlorophyll under high light treatment, we also found the significant change of chlorophyll a fluorescence parameters between 200 and 400 µmol m^−2^ s^−1^ light intensities. Usually, 1000 µmol m^−2^ s^−1^ light intensities causes photoinhibition in plants, and results in the increase of Fo and decrease of Fv/Fm [23]. In rubber tree clone GT1 leaves, Fv/Fm and Fv/Fo kept constant at different light intensities, which was in accordance with the previous study [24]. The stable Fv/Fm indicated no damage to the structure and function of chloroplast. The great divergence of Y(II), qP, ETR, and qL suggested both the donor and acceptor side of photosynthetic transfer chain could not use excite light energy effectively at 400 µmol m^−2^ s^−1^ light intensity. These light energy were quenching through Y(NO) (Figure 3). High values of Y(NO) indicated that excess excitation energy was mostly channeled off via basal quenching mechanisms and, hence, energy fluxes were inadequately controlled.

ROS play dual roles in plant stress response [25]. It's related to the functions of mitochondria [26], [27], chloroplasts [28], and peroxisome [29]. H_2_O_2_ content was significantly increased with the increase of light intensity (Figure 5). The SOD and POD enzyme activities showed decrease patterns. Excessive light energy caused the increase of ROS but did not exceed the endurance of rubber tree leaves. So no damage caused to the performance of PSII under high light irradiation in rubber tree. Gene expressions safely explained the effects of high light irradiation on the mitochondria and chloroplasts functions and antioxidant enzymes activities. The peroxidase, catalase, and SOD genes expressions were highest under 400 µmol m^−2^ s^−1^ light intensity (Figure 8). These indicated that more antioxidant enzymes were formed to cope with the decreases of per unit activity of enzymes and increase of ROS, and avoid membrane lipid peroxidation. The early response of *HbATP*, *HbRbsB*, and *HbCAX* contributed to the increase of photochemical quenching under 200 µmol m^−2^ s^−1^ light intensities (Figure 2). These genes were participated in energy formation, defense response, and chloroplast development. ASRs have been reported to be members of the widespread class of hydrophilins, including the seed-specific late embryogenesis abundant (LEA) proteins [30]. Light inhibited *HbASRLP1* gene expression, suggesting that unlike drought stress, light did not induce hydrophilins production in rubber tree. These results indicated several signal pathways harmoniously involved in response to changes of light intensities. It seemed that light up-regulated expression of light responsive genes and down-regulated expression of genes involved in other signal pathways, such as drought stress.

Taken together, it's safely assumed that 200 µmol m^−2^ s^−1^ light intensities would be an appropriate light condition for breeding seedlings of rubber tree clone GT1. 400 µmol m^−2^ s^−1^ light intensities induced the adjustment of mitochondria and chloroplasts by regulating genes expressions of antioxidant enzymes and enzymes construction to avoid potential damages to plant. The tropical plant rubber tree could endure strong light irradiation via a specific mechanism. Adaptation to high light intensity is a complex process by regulating antioxidant enzymes activities, chloroplast formation, and related genes expressions in rubber tree.

## Materials and Methods

### Plant materials and growth conditions

Rubber tree clone GT1 (original clone breed in Indonesia) seedlings were grown in the plastic pots in the chamber with vermiculite and turfy soil (1∶3) at the experimental farm of the Chinese Academy of Tropical Agricultural Sciences in Danzhou city, Hainan province, China (19°51′51N; 109°55′63E). In growing season, the average temperature was about 30°C, precipitation was about 180 mm, and humidity was around 97.5%. Leaves samples in dark green stage were collected from seedlings under the indicated light intensity treatment provided by a 1000 W tungsten bulb. A water tank with recycled water was used between radiation source and samples to absorb heat.

### Measurment of chlorophyll content

Chlorophyll was extracted with 80% ice cold acetone from 0.1 g leaves sample. The extract was measured at 475, 645, and 663 nm with spectrophotometer (General Electric, Fairfield, CT, USA), respectively. Specific chlorophyll and β-carotene contents were determined according to the reported method [31].

### Modulated chlorophyll a fluorescence measurment

Modulated chlorophyll a fluorescence measurement was made in attached leaves at mid-day with a PAM-2500 portable fluorometer (Walz, Effeltrich, Germany) connected to a computer with data acquisition software Pam-Win3 (Heinz, Walz). The experimental protocol described by [19] was essentially followed. The minimal fluorescence level (F_0_) in dark-adapted state was measured by the measuring modulated light, which was sufficiently low (<0.1 µmol m^−2^ s^−1^) not to induce any significant variable fluorescence. To determine the minimal fluorescence level during illumination (F_0_′), a black cloth was rapidly placed around the leaf and the leaf-clip holder in the presence of far-red light (7 µmol m^−2^ s^−1^) in order to oxidize the PSII center fully. Upon darkening the leaf, fluorescence dropped to the F_0_′ level and immediately rose again within several seconds. The maximal fluorescence level in the dark-adapted state (F_m_) and the maximal fluorescence level during natural illumination (F_m_′) were measured by a 0.8-s saturating pulse at 8000 µmol m^−2^ s^−1^. F_m_ was measured after 30 min of dark adaptation. F_m_′ and F_s_ were measured when photosynthetic photon flux densities (PPFDs) were approximately 200 and 1400 µmol m^−2^ s^−1^, respectively. Other parameters were calculated based on measured parameters above.

### Measurement of MDA content

MDA content was determined by the thiobarbituric acid (TCA) reaction as described by [32]. 1 g fresh weight of leaves sample was homogenized in 5 ml 0.1% (w/v) TCA. The homogenate was centrifuged at 10000 g for 5 min and 4 ml of 20% TCA containing 0.5% (w/v) thiobarbituric acid (TBA) was added to 1 ml of the supernatant. The mixture was heated at 95°C for 30 min and then quickly cooled on ice. The contents were centrifuged at 10000 g for 15 min and absorbance of the supernatant at 532 and 600 nm was recorded. After subtracting the non-specific absorbance at 600 nm, the MDA concentration was determined by its extinction coefficient of 155 mM^−1^ cm^−1^.

### Measurements of activities of SOD and POD, and H_2_O_2_ content

SOD (EC 1.15.1.1) was prepared by first freezing 0.5 g of leaves sample in liquid nitrogen to prevent proteolytic activity, followed by grinding with 5 ml extraction buffer (0.1 M phosphate buffer, pH 7.5, containing 0.5 mM EDTA and 1 mM ascorbic acid). Brie was centrifuged for 20 min at 15000 g and the supernatant was used as an enzyme. The soluble proteins concentration in the supernatant were determined using the method of Bradford with bovine serum albumin (BSA) as standard [33]. The per unit activity of SOD was estimated by recording the decrease in optical density of nitro blue tetrazolium (NBT) induced by the enzyme [34]. 3 ml of the reaction mixture contained 13 mM methionine, 75 µM nitroblue tetrazolium chloride, 0.1 mM EDTA, 50 mM phosphate buffer (pH 7.8), 50 mM sodium carbonate, and 0.1 ml enzyme solution. The reaction was started by adding 2 µM riboflavin. The reaction mixtures were illuminated for 15 min at 90 µmol m^−2^ s^−1^ (placing the test tubes under two 15 W fluorescent lamps). A complete reaction mixture without enzyme, which gave the maximal colour, was served as the control. The reaction was stopped by switching off the light and putting the tubes into dark. A non-irradiated complete reaction mixture was served as a blank.

POD (EC 1.11.1.7) activity was determined with spectrophotometer. 0.5 g leaves sample was extracted with 5 ml 100 mM phosphate buffer (pH 6.0). Homogenate was centrifuged at 4000 g for 10 min. Reaction mixture was 50 ml 100 mM phosphate buffer (pH 6.0) with 23 mM guaiacol and 1.8 mM hydrogen peroxide. 1 ml supernatant was added into 3 ml reaction mixture. The change of OD was recorded at 470 nm. The per unit activity of enzyme was defined as the increase of 0.1 ΔOD per minute.

Measurement of H_2_O_2_ content was conducted according to the reported method [35]. Leaves were ground into powder in liquid nitrogen. Weighed 0.5 g of the powder, put into the 10 ml centrifuge tube by adding 2 ml pre-cooled acetone. Mixture was vortexed and placed in an ice box extracting about 30 min. After extraction, the solution centrifuged at 10000 g for 10 min. 1.5 ml of the supernatant was put into a new 15 ml centrifuge tube and added 3 ml extract liquid (CCl4: CHCl3 = 3: 1, v: v), shaking vigorously. Added 5 mL ddH_2_O, shaken vigorously again and placed in an ice box for 5 min. After stratification of liquid solution, the tube centrifuged at 6500 g for 5 min. Took 5 ml of the upper aqueous phase, added 0.5 mL 5% TiSO_4_ and 1 ml of concentrated ammonia water in a centrifuge tube. Shaking mixture to make precipitation with ample color, centrifuged at 10000 g for 10 min, discarded supernatant. The deposition was dissolved by 5 ml of 2 M H_2_SO_4_, and filtered with filter paper. Filtrate was measured OD at 415 nm with spectrophotometer.

### Gene expression analysis by real-time PCR

Total RNA was extracted from leaves according to the methods of Tang et al. (2007) and Xiao et al. (2009) [36], [37]. The quality and concentration of the extracted RNA were detected by agarose gel electrophoresis and measured by a spectrophotometer.

First strand cDNA was synthesized from 2 µg of total RNA with MMLV reverse transcriptase and random hexamer primer (Takara) according to the manufacturer's instruction. The cDNA was diluted 1∶20 with nuclease-free water. Aliquots of the same cDNA sample were used for real-time PCR with primers designed for the selected genes, and *18S rRNA* (*Hb18SRNA*) was used as a house-keeping gene (Table 1). The PCR reaction was performed in a 20 µL reaction mixture containing 200 nM of each primer, 1×SYBER Green PCR Master Mix (Takara), and about 30 ng cDNA. Real-time PCR was performed on the LightCycler 2.0 system (Roche Diagnostics, Germany). The program used was as follows: 30 s at 95°C for denaturation; followed by 40 cycles of 5 s at 94°C, 30 s at 60°C, and 40 s at 72°C. The relative abundance of transcripts was calculated according to the LightCycler relative quantification software 4.05 instructions. The specificity of each primer pairs was verified by determining the melting curve at the end of each run and by sequencing the amplified bands from gel electrophoresis.

**Table 1:** Information of primers used in this study.

Genes	Accession	Primer sequences (5′-3′)	Amplification	Amplification	Reference
	Number		length (bp)	efficiency	
*HbCOA*	AY461413	Forward: GGTGACATGGTGGTGAAT	145	1.872±0.0183	[11]
		Reverse: TGAAGTGACGAATGAGGTAA			
*HbCOAL*	AF429387	Forward: GAGTATCCAGTTAGGCATCA	119	1.918±0.0099	Direct
		Reverse: CTAGTGAATCATGTCCAAGTC			submission
*HbAPX*	AF457210	Forward: CCAACTGACACCGTTCTT	164	1.815±0.0067	[12]
		Reverse: CAGCACCATCCTCTACATC			
*HbASRLP1*	AY221984	Forward: GGAAAGAAGCACCACCAT	125	1.868±0.0114	Direct
		Reverse: TACAGAGATGAACAGGCAAT			submission
*HbATP*	X58498	Forward: GCTTCACGCAGACTATTATC	112	1.809±0.0079	[13]
		Reverse: TAGAGGATGGAGATGAGGAA			
*HbCAT*	AF151368	Forward: GGTATTGTGGTTCCTGGTAT	153	1.877±0.0093	Direct
		Reverse: ATGGTGATTGTTGTGATGAG			submission
*HbCBP*	X89855	Forward: GTTGCCACAGACATCTACA	113	1.891±0.0062	[38]
		Reverse: TCATCATTGACCACCAGTT			
*HbCOXI*	AJ223436	Forward: AAGACACAATCAGCAGGTAA	102	1.973±0.0063	[39]
		Reverse: TAGCCACTCACAGAAGGAT			
*HbCAX*	AY207389	Forward: CTGCTGCTGAATCTCCTT	167	1.937±0.0021	[40]
		Reverse: CTCTTGCTTGACGGCTAT			
*HbCuZnSOD*	AF457209	Forward: GTCCAACCACCGTAACTG	200	1.901±0.0109	Direct
		Reverse: GCCATCATCACCAACATTG			submission
*HbMnSOD*	L11707	Forward: TGTGCTGTAATGTTGACCTA	128	1.873±0.00139	[41]
		Reverse: GTTCACCTGTAAGTAGTATGC			
*HbRbsS*	M60274	Forward: GCCAAGGAAGTTGAATACC	123	1.794±0.0256	[42]
		Reverse: CCAGTAACGACCATCATAGT			
*Hb18SRNA*	AY435212	Forward: GCTCGAAGACGATCAGATACC	146	2.062±0.011	Direct
		Reverse: TTCAGCCTTGCGACCATAC			submission

### Statistical Analysis

All data were analyzed with IBM-SPSS analytical software package version 20.0 (IBM Corporation, USA). One-way ANOVA and Tukey text were used to assess the different level. *P*<0.05 (probability level) was considered significant difference. Figures were drawn by Origin data analysis and graphing software, Origin Pro 9.0 (OriginLab Corporation, USA). For real-time PCR analysis, each value was the average of two biological replicates tested in triplicate, and for other analysis, 6 replicate samples tested in replicate were used.
